# Recurrent Transformation of Prior Knowledge Based Model for Human Motion Recognition

**DOI:** 10.1155/2018/4160652

**Published:** 2018-01-14

**Authors:** Cheng Xu, Jie He, Xiaotong Zhang, Haipiao Cai, Shihong Duan, Po-Hsuan Tseng, Chong Li

**Affiliations:** ^1^School of Computer and Communication Engineering, University of Science and Technology Beijing, China; ^2^Beijing Key Laboratory of Knowledge Engineering for Materials Science, Beijing, China; ^3^Department of Electronic Engineering, National Taipei University of Technology, Taipei, Taiwan; ^4^The Computer Network Information Center (CNIC), The Chinese Academy of Sciences (CAS), Beijing, China

## Abstract

Motion related human activity recognition using wearable sensors can potentially enable various useful daily applications. So far, most studies view it as a stand-alone mathematical classification problem without considering the physical nature and temporal information of human motions. Consequently, they suffer from data dependencies and encounter the curse of dimension and the overfitting issue. Their models are hard to be intuitively understood. Given a specific motion set, if structured domain knowledge could be manually obtained, it could be used for better recognizing certain motions. In this study, we start from a deep analysis on natural physical properties and temporal recurrent transformation possibilities of human motions and then propose a useful Recurrent Transformation Prior Knowledge-based Decision Tree (RT-PKDT) model for recognition of specific human motions. RT-PKDT utilizes temporal information and hierarchical classification method, making the most of sensor streaming data and human knowledge to compensate the possible data inadequacy. The experiment results indicate that the proposed method performs superior to those adopted in related works, such as SVM, BP neural networks, and Bayesian Network, obtaining an accuracy of 96.68%.

## 1. Introduction

Human motion related activity recognition (HAR) is one of the most promising research topics for a variety of areas and has been drawing more and more researchers' attention. With the booming of Internet of Things (IoTs), sensors have been widely used in HAR applications, due to the advantages of no need to deploy in advance, smaller data volume, lower cost, and power consumption. Sensors-based HAR stands out among various technologies [1–3] and has been drawing tremendous attention and applied into a variety people centric application areas, such as medical care [1], emergency rescue [2], and smart home surveillance [3].

However, obtaining sufficient information from sensor data sequences to recover the parameters of body motion correctly is a challenging task for two reasons. The first is the large number of degrees of freedom in human body configurations, resulting in high computational loading, and the second is the large variability and uncertainty in motor movements employed for a given motion.

To solve the first problem, most related works use data-driven methods which tend to take the advantage of multiple sensors [4], such as accelerometer, gyroscope, compass sensor, and humidity sensor, to name but a few, to enlarge the input data set to achieve more information. More than one sensor node is mounted onto different body-parts to monitor human motions with multiple degrees of freedom. In [5], Stiefmeier studied how sensors bounded to different body-parts, such as Torso, sleeve, arm, and hand, contribute to the recognition of complex human motions. Above methods somehow expand the data source; however, the introduction of redundant data may not only lead to extra burden on computational capability, but also cause dimension disaster problem [6] which on the contrary degrades the classifier's performance. Data-driven methods hardly look into the nature of motions and extract most important features by empirical analysis or engineering methods [7, 8]. To solve this problem, more attention should be paid to focus on the physical nature of human motion characteristics and filter key information for recognition. Ghasemzadeh and Jafari [8] introduce a novel classification model that identifies physical movements from body-worn inertial sensors while taking collaborative nature and physical combinations of different body joints into consideration. With physical information, [8] maintains 93.3% classification accuracy.

To solve the second problem, probability and statistics methods are introduced to overcome human motion's uncertainty. HMM [13] and Bayesian Network [7] are the most widely considered algorithms to solve this problem. Bayesian Network can cope with uncertainty, erroneous or missing sensor measurements. Despite the fact that these classifiers assume conditional independence of the features, the classifiers yield good accuracy when large amounts of sample data are provided. The hidden Markov model (HMM) is probably the most popular generative approach that includes temporal information. An HMM is a probabilistic model with a particular structure that makes it easy to learn from data, to interpret the data once a model is learned, and is both easy and efficient to implement. Bayesian Network and HMMs form the basis of statistical temporal models; however, model for each certain activity should be modeled and prior probability should be prepared before model is trained. However, accurate probability is difficult to be obtained due to the complexity and subjectivity of human motions, as well as the requirement of large amounts of actual data. Motions are performed under different environments simultaneously, such as applications in medical care and emergency rescue [1, 2, 14].

Data-driven methods may cover most applications but they may be not suitable for some specific scenarios. As Bousquet stated in [15], specific knowledge can help improve generalization performance. Correspondingly, knowledge-driven methods are more suitable for applications with specific backgrounds, namely, direct human knowledge. Knowledge-driven activity recognition is founded upon the observations that most activities, in particular, take place in a relatively specific circumstance of time, location, and space. Knowledge-driven activity modeling and recognition intend to make use of rich domain knowledge and heuristics for activity modeling and pattern recognition [16]. The rationale is to use various methods, in particular, knowledge engineering methodologies and techniques, to acquire domain knowledge. Comparing with data-driven activity modeling that learns models from large-scale datasets and recognizes activities through data intensive processing methods, knowledge-driven activity modeling avoids a number of problems, including the requirement for large amounts of observation data, the inflexibility that arises when each activity model needs to be computationally learned, and the lack of reusability that results when one person's activity model is different from another's [16].

For particular applications, target motion set is generally fixed and structured domain knowledge could be manually obtained and utilized for better recognizing certain motions. Motions or activities are completed in a certain sequence. These rules could be obtained in advance, and we may use these relations to help recognize the activity. In these conditions, prior knowledge can enlighten the human activity recognition on the basis of data-driven methods.

In this paper, we put forward a sequential recognition method RT-PKDT (Recurrent Transformation Prior Knowledge based Decision Tree) to recognize human motion related activities, with consideration of a conceptual model. By deeply mining commonly understanding motions, a conceptual motion model is considered. Temporal information is considered and a recurrent transformation method is put forward to realize sequential human motion recognition. With applying RT-PKDT into motion classification and the integration of Support Vector Machine (SVM) using RBF Kernel, it improves the classification performance and makes up for the inadequacy of data itself. Result shows that our proposed method works better than traditional methods such as SVM, BP, and Bayesian Network and has achieved a general true classification rate of 96.68%.

## 2. Construction of PKDT

Prior knowledge plays a big role in the whole classification process. To solve aforementioned problem, we try to bring more expert knowledge into the classifier to achieve the goal of extracting and using key features to improve classification performance in the motion recognition process. In this section, we present a new approach, prior knowledge based decision tree (PKDT), by exploring rich domain knowledge for activity classification rather than learning them from data as seen in data-driven approaches.

As there may be lots of different activities in daily life and we cannot take all into consideration, we turn to the most frequently appearing motion for medical care and emergency rescue scenario including Standing, Lying, Walking, Running, Walking upstairs, Walking downstairs, elevator up (short for upstairs by elevator), and elevator down (short for downstairs by elevator). The activity case set can be given by

(1) Activity=Standing (St), Lying (Ly), Walking (Wa), Running (Ru), Upstairs (Up), Downstairs (Do), ElevatorUp (Eu), ElevatorDown (Ed)

### 2.1. Conceptual Motion Model

**Figure 1:**
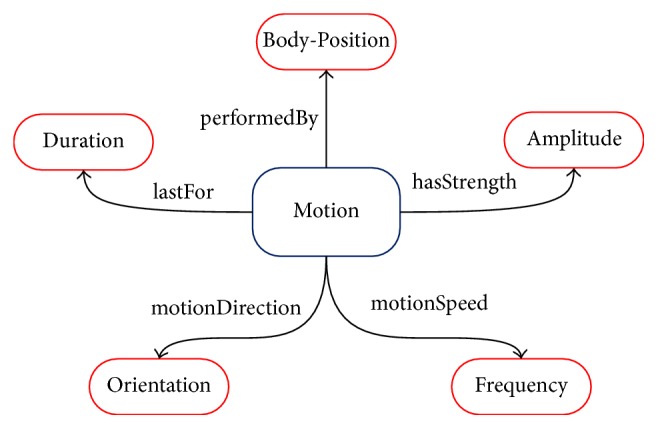
The conceptual motion model. Each motion can be viewed as a combination of five attributes: intensity, orientation, velocity, body-position, and duration.

**Figure 2:**
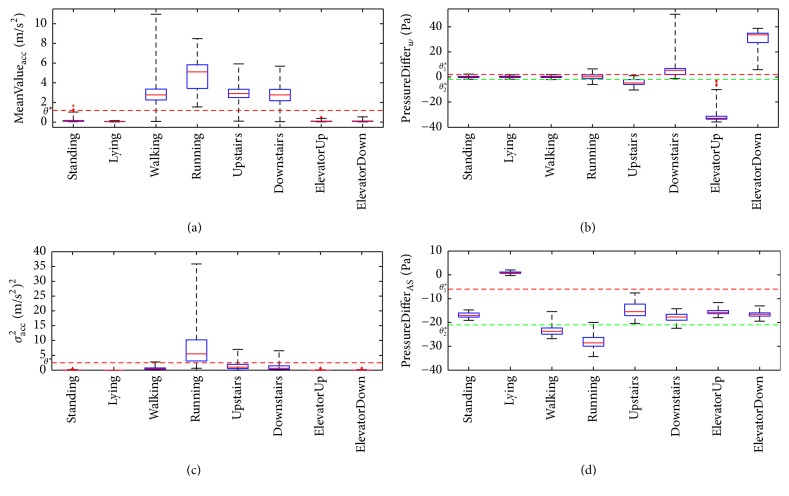
Boxplot of four features corresponded, respectively, to the attributes demonstrated in motion model. Typical features corresponded, respectively, to the attributes demonstrated in motion model and are calculated based on collected dataset. (a) is based on mean value of acceleration; (b) is based on the difference of pressure measurement value in a given time window; (c) is based on the variance of the acceleration; (d) is based on the pressure difference between Ankle and Shoulder.

As for activity recognition problems, prior knowledge is reflected in our understanding of motions. It is commonly believed that a human motion can be described from several attributes, like intensity, orientation, velocity, and so on. These attributes, in some aspects, embody characteristics of motions and can be related to a series of key features that most eminently reflect the physical difference among activities. These key features may be used to group different kinds of activities into several subclasses as they have various distribution overlap on the same attribute. We thus make the most of the common sense knowledge exploring the physical attributes of daily human motions to construct a conceptual motion model, as shown in Figure 1. We model a human motion with attributes of intensity, orientation, velocity, body-position, and duration. Each attribute represents human motions in a side view from a particular angle. Detailed explanation and analysis are described as follows:

- *Intensity*: different motions behave differently in the performance of exercise intensity. In everyday life, activities, such as Walking, Running, Walking upstairs, and Walking downstairs, consist of a series of periodic mechanical actions, while activities, such as Standing, Lying, ElevatorUp, and elevatordown, are almost relatively static to surrounding environment. Therefore, taking the difference of intensity attribute between different activities, we can divide the activity case set into two subclasses, the former* Active activity* and the latter* Rest activity*. Features, like mean value of acceleration (*MeanValue*_*acc*_, shown in Figure 2(a)) are to some extent related to activities' intensity attribute. Distinction between active and rest activities can be easily made with the use of intensity related features.
- *Orientation*: movements' orientation is also one of the most intuitive attributes in common knowledge sense. As terrestrial reference coordinate system is often thought of as the default coordinate system, everyday activity can be classified into two subclasses: (1)* Vertical Motion*, including {WalkingUpstairs, WalkingDownstairs, ElevatorUp, ElevatorDown}, and (2)* Horizonal Motion*, including {Standing, Lying, Walking, Running}. The pressure value got from barometer sensors directly reflects the characteristics and differences between them. Features extracted from pressure value, such as the difference of pressure measurement value in a given time window (*Pressure*_*w*_, shown in Figure 2(b)) intuitively show how pressure, namely, height, changes over time.
- *Velocity*: velocity can clearly and effectively describe how fast humans repeat the motion. Considering the obvious differences among activities with different motion velocity, we can group activities into* Relatively High Velocity Motion* and* Relatively Low Velocity Motion*, taking Running and Walking as an example. And it also works on WalkingUpstairs (or WalkingDownstairs) versus ElevatorUp (or ElevatorDown). Features like variance of the acceleration (*σ*_acc_^2^) and mean crossing rate of acceleration and gyroscope (*MCR*_*acc*_) reflect sensor data's vibration with the going of activity.
- *Body-position*: human activities can be seen as a combination of a series of body-part movements instead of being performed by one single body-part, which means distinction may arise from body-position where sensors are mounted. In other words, for certain activities, it may have similar distribution of sensor data from one body-part, while clearly difference will be seen when several body-parts' data distribution is viewed together, which can be made use of to do the distinction. For example, Standing and Lying are two static activities while sensors on single body-part are almost invariable. It is very difficult to separate them from each other with data from only one body-part. However, if data from sensor mounted to Ankle and Shoulder are combined, the pressure difference between these two position* (PressureDiffer*_*AS*_) will contribute greatly to the distinction of the two activities.
- *Duration*: every activity lasts for a certain time, and it is easy to be understood that a reasonable time window is necessary to better distinguish activities. If we certainly know how long a particular activity lasts for, we could obtain more useful information with the help of analyzing the whole activity process. Previous researches are not unified on determination of the time window length which is already discussed in Section 2. In this study, we take an empirical window length of 2 seconds, in order to avoid the complexity of the problem and improve the classifier's generalization performance.

The above attributes constitute various activities. One feature may work towards the classification process based on one attribute but may not towards another. Purpose of the study in this paper is to make the most of the differences among activities' attributes in order to tell them apart. Therefore we explore the rich common knowledge extracting the key features to construct a prior knowledge based decision tree model with analyzing attributes' distribution in methods detailed in next section.

### 2.2. Prior Knowledge-Based Decision Tree

The proposed conceptual model above establishes links between activities and conceptual information through activity-based attributes and makes it possible to understand and distinguish different motions in finer perspectives. At the same time, multiclass classification could be done in steps one of which adopts one attribute as a basis. In this way, hierarchical relationships are constructed that link conceptual information with sensor observations through activity attributes. Above-mentioned considerations similarly make decision tree classifier a first choice with the advantage of easier to build multilevel heuristic structure as decision tree is a set of if-then rules which are successively applied to the input data. Based on the analysis of activity attributes, we propose a fusion method, Prior Knowledge-based Decision Tree (PKDT), to achieve the goal of classification in a hierarchical way which at the same time pursues a better generalization performance.

**Figure 3:**
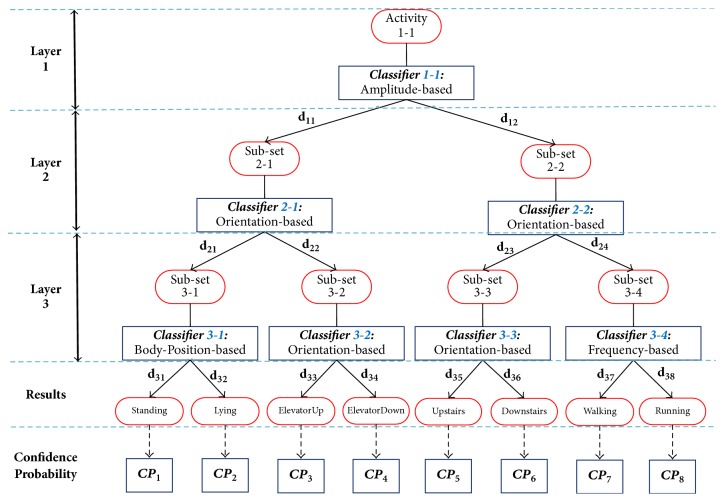
Prior Knowledge-based Decision Tree: A typical classification method according to commonly human sense.

Making use of the characteristics of different attributes, a typical heuristic decision tree based classification model is demonstrated in Figure 3. In this binary tree structure, each internal node is replaced with an activity attribute related binary classifier, so as that a multiclassification problem transforms into multiple binary classification problem which can make the most use of balanced binary tree and internal binary subclassifiers.

Support Vector Machine (SVM) [17] is selected as internal classifier and it may work out the confidence probability (CP) of each candidate classes via decision values [17], denoted as $\vec{d}$. The class with the maximum probability is considered to be the estimated result. For a SVM classifier intending to classify *N* classes, it may give out the decision value of each classifier, which can be mapped to confidence probability by activation function, namely,

(2) $$\begin{array}{c} CP=f\left(\;\vec{d}\;\right)=\frac{1}{1+\exp\left(-\vec{d}+1\right)}. \end{array}$$

As demonstrated in Figure 3, our proposed PKDT has 3 layers which have 2^*i*^ − 1 internal classifiers in the *i*th layer. In the *i*th layer, the input instance are further classified into 2^*i*^ subclasses. The *j*th classifier in the *i*th layer, whose discrimination function is *g*_*i*,*j*_(**x**^*t*^∣*θ*), gives out decision values for internal classification results. Decision values generated in the *i*th layer could be denoted as $\vec{d_{i}}$, while $\vec{d_{i}}=[d_{i,1},\ldots ,d_{i,m}]$, *m* = 2^*i*^. In bottom layer, final decision values *d*_*k*_ for the *k*th candidate motion are achieved via multiplicative *d*_*k*_ = ∏_*i*=1_^3^*d*_*i*,*k*_, *k* = 1,…, 8. For a specific instance *x*^*t*^ at time *t*, confidence probability of the *k*th human motion is CP_*k*_ (mapped with *f*(*d*_*k*_)). The classification result (*R*_*t*_) is represented with the maximum CP and worked out by intermediate results *d*_*k*_ as shown in

(3) $$\begin{array}{c} R_{t}=argmax\left(\;CP_{k}\;\right),\;k=1,2,\ldots ,N, \end{array}$$

where *R*_*t*_ is the classification result with the maximum confidence probability, ranging from 1 to 8 as there are 8 candidate human motions.

Based on the aforementioned fusion method, with the advantages of hierarchical display, a balanced binary decision tree is constructed in which each internal node is replaced with an activity attribute-based binary subclassifier. It is worth stressing that the five attributes of motion may make no identical contribution on the activity classification so that there could be a particular combination method of these attributes used in PKDT. Among the five attributes mentioned above, Duration is viewed as a fixed parameter in this study. Intensity and Orientation are of certain indicators that can separate one from the others, while Frequency and Body-Position attribute have the nature of relativity which makes them only suitable for local distinguish rather than global distinguish. Taking another reason into consideration, attribute with the largest classification performance should be placed in the root classifier in order to get a better result along with the latter classification process. By practical validation, the demonstrated structure is the most effective one.

In PKDT method, a knowledge-driven recognition path flows from the root node to leaf node, passing by activity attribute related internal classifier. In this way, the overfitting problem can be to some extent avoided. However, temporal information is not yet considered and, in some conditions, relationship between layers could be utilized for computational reduction.

## 3. Recurrent Transformation Model

A complex human motion typically consists of multiple primitive events happening in parallel or sequentially over a period of time. Understanding such complex motion requires recognizing not only each individual event but also, more importantly, capturing their temporal dependencies. This is in particular the case when the detection of individual events is poor due to poor tracking results, occlusion, background clutter, and so on. In this section, the transformation relationship between various human motions is studied and we propose an hierarchical recurrent transformation model for human motion recognition.

The model is constructed via two considerations: human motion's physical attributes and temporal transition dependencies among human motions. Since the PKDT has already considered physical information, in this section we mainly introduce how temporal information could be included in the motion classification process.

### 3.1. Temporal Transition Model

We now give a formal description of an sequential transformation human motion. Let Σ be a finite alphabet, each element *O* of which stands for a single motion. We denote by Σ^*∗*^ the set of all possible strings over Σ. An observation sequence of human activity is a finite string from Σ^*∗*^ denoted by $\bar{O}=o_{1}o_{2}\cdots o_{T}$. These temporal transition constraints between different motions are acquired by statistics in HMM and Bayesian Network methods [7, 13].

**Figure 4:**
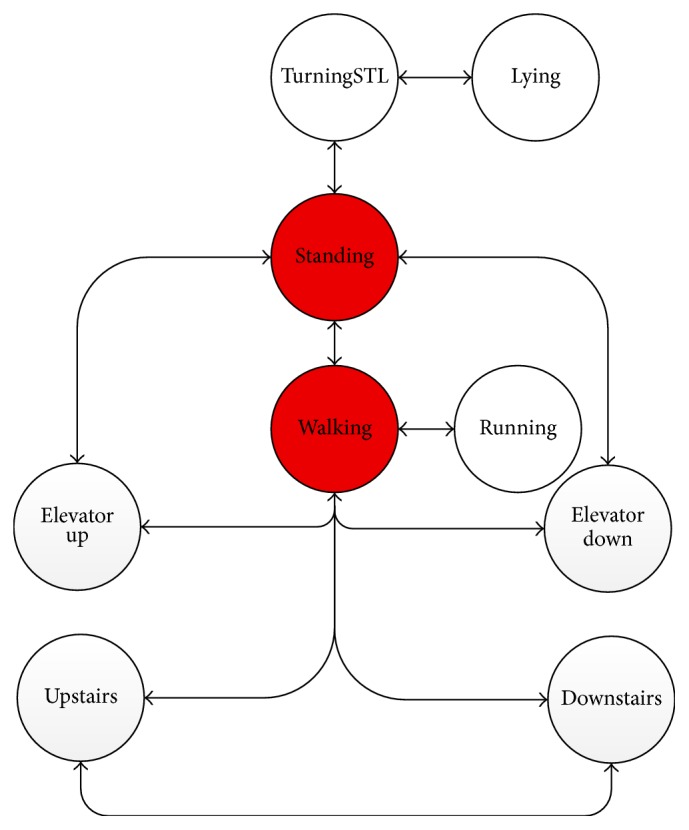
Conceptual transformation relationship between human motions listed in activity.

However, in practice these probabilities are hardly available because human motions are often stochastic and paroxysmal. With this taken into consideration, we take human knowledge as constraints other than statistical probabilities. In human common sense, there should be causal connections between motions. For example, after Running there should be a “Walking” for a period of time; then it may come to “Standing” or perhaps “Running” again. However, it is unreasonable that “Lying” immediately comes after “Running” (do not take falling into consideration, as there at least is a conversion process). Without being very particular, it may be unreasonable to suddenly change from “Lying” to “Downstairs.” Figure 4 simply shows possible transition relationship according to human sense, in which each arrow represents possible transitions between daily human motions.

With these cognitive constrains, more accurate pattern recognition could be realized and it will be shown in the following studies. All these possibilities and impossibilities could be inducted as shown in Figure 4, according to human prior knowledge. Detailed transition relationship is demonstrated in Table 1, where “1” stands for transferrable and “0” stands for nontransferable.

CP is confidence probability of activity classification, which could be achieved from SVM classifier [17]. Trans1(*t* − 1, *t*) is the transition matrix which indicates the possible transitions between time *t* − 1 and time *t*. The expected output *R*_*t*_ is the classification result with the maximum confidence probability. In consideration of last time recognition result *R*_*t*−1_, the constraints described in Table 1 are contained in transition matrix Trans1(*t* − 1, *t*), and the supposed impossible transition is limited to 0 as the confidence is set as 0. By this means, a classification process is completed at certain time *t*.

**Figure 5:**
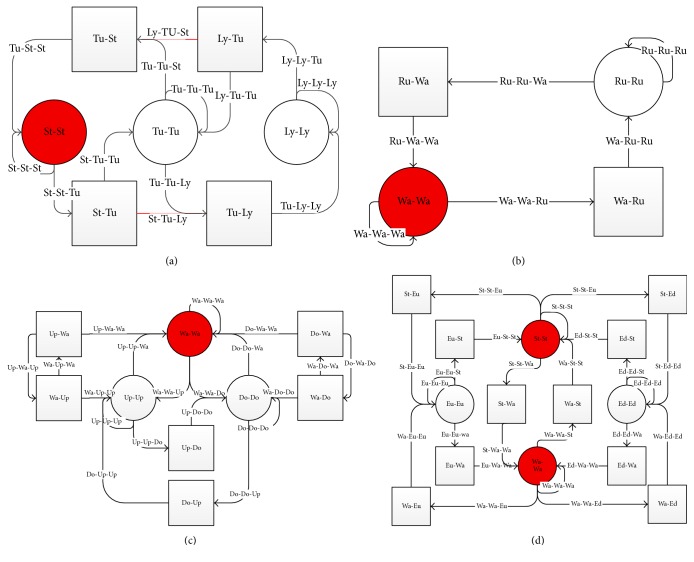
Second-order transition schematic diagram. The possible transferability between motions with the constraints is demonstrated. (a) demonstrates the possible transitions among activities* Standing*,* TurningSTL,* and* Lying*. (b) demonstrates the possible transitions among activities* Walking* and* Running*. (c) demonstrates the possible transitions among activities* Walking*,* Upstairs,* and* Downstairs*. (d) demonstrates the possible transitions among activities* ElevatorUp*,* Elevatordown*,* Standing,* and* Walking*.

Furthermore, apart from the transferability, the temporal connection between motions should be also taken into classification process. For facilitating the description, we model the possible transferability between motions with the constraints demonstrated in Table 1. Possible transitions are judged by common prior knowledge and do not depend on data acquisition and statistics in advance. It could be viewed as a simplified Markov model in which transition probabilities are set to “0” or “1.” For motion *R*_*t*_ at a given time *t*, its former motion state *R*_*t*−1_ is considered. With the truth Table 1, some unreasonable transitions are ruled out, and possible transitions are shown in Figure 5. These possible transitions are drawn by lines, while transition is unreasonable to common sense where these is no line drawn between states. Particularly, two red lines are drawn in Figure 5, which means an intermediate state (“Standing” to “Lying” or “Lying” to “Standing”) is separately considered as the process is relatively long compared with other motions.

However, there may still exist some problems. In some conditions, given a prior state *R*_*t*−1_, the possible estimated result of next state is constrained within a certain range. Current human motion is clearly related to historical motions within a time window. Methods mentioned above merge human knowledge of possible transitions into classification process; however, temporal information is not being fully exploited. More historical information can be added to the classification process.

For the sake of this, a second-order transition model is proposed as shown in Figure 5. Prior knowledge is considered that for a certain time *t*; its current state is directly related with both the last state and the next possible state, namely, states at time *t* − 1 and *t* + 1. Possible second-order transitions between human motions are described in Figure 5. Similarly, a second-order transition matrix Trans(*t* − 1, *t*, *t* + 1) could be derived, which could be easily calculated if Trans1(*t* − 1, *t*) is maintained well. Their relationship could be represented as

(4) Transt−1,t,t+1=Trans1t−1,t∗Trans1t,t+1,

where Trans1(*t* − 1, *t*) = Trans1(*t*, *t* + 1). Namely, the second-order transition matrix is the square of first order matrix. Unreasonable judgements are ruled out with second-order transition matrix considered. It is worthy to mention that the more temporal information considered, the better recognition result could be got. But the conceptual model would be rather complex as the second-order model is already complicated. So only second transition model is adopted. The recognition target could then be updated as

(5) Rt=argmaxCP1CP2⋯CPN∗Transt−1,t,t+1.

### 3.2. Recurrent Prior Knowledge Based Decision Tree

**Figure 6:**
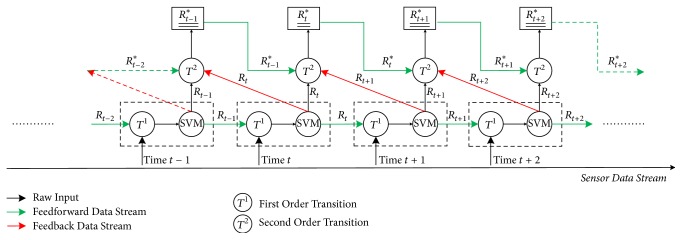
RT-PKDT. At certain time *t*, the classification process is proceeded by PKDT method.

With sequential transition relationship being ruled as shown in Figure 5, recognition could be realized with adding these rules into PKDT method. Combined rules may correct some misclassification results when transition information is not taken into consideration. Then a recurrent transition prior knowledge-based decision tree method (RT-PKDT) is proposed. This hierarchical rules constrained method utilizes the temporal information between motions together with hierarchical classification decision tree, the model of which is shown in Figure 6.

RT-PKDT synthesizes the advantages of hierarchical classification and temporal transition method. It is human readable and combines the prior knowledge in the classification process and at the same time takes human motion's temporal characteristics into consideration. As shown in Figure 6, at certain time *t*, the classification process is proceeded by PKDT method. The classification process could be divided into the following three steps: at time *t*:

1. Raw data is processed in the first place, extracting and selecting features. Motion transition bounds demonstrated in Figure 4 are considered. The integration embodies in the use of result at time *t* − 1 with first order transition matrix *T*^1^. By this constraint, unreasonable states are ruled out and further classification is done by PKDT.
2. With PKDT structure, confidence probability matrix is worked out. The target motion with maximum CP is selected as candidate result *R*_*t*_.
3. The same operation above is proceeded again at time *t* + 1 and result *R*_*t*+1_ is achieved. Then, result at time *t* is updated with second-order transition matrix, representing (5). Final classification result is got which is represented as *R*_*t*_^*∗*^.

By the above process we can see that, in RPKDT method, final result *R*_*t*_^*∗*^ is bounded to the last time (time *t* − 1) result *R*_*t*−1_ and the next time (time *t* + 1) result *R*_*t*+1_.

### 3.3. Feature Selection

In order to have more flexibility and have a better description on the classification ability of different features, we bring in a quantification mechanism, with which the best combination of features needed by each subclassifier is extracted. Detailed algorithm will be demonstrated as follows.

#### 3.3.1. Feature Quantification

As analyzed above, a key feature should have a less distribution overlap so we bring in the conception of* Divergence* [9] to quantize class separability. While the ratio *P*(**x**^*t*^∣*A*_*i*_, *θ*)/*P*(**x**^*t*^∣*A*_*j*_, *θ*) can reflect the distinguishing capability of feature vector **x**^*t*^ on activity *A*_*i*_ and *A*_*j*_, divergence [9] can be denoted as

(6) dij=Dij+Dji=∫−∞+∞Pxt ∣ Ai,θ−Pxt ∣ Aj,θ·ln⁡Pxt ∣ Ai,θPxt ∣ Aj,θdxt

and one feature's* AverageDivergence* is denoted as

(7) $$\begin{array}{c} \bar{d}=\overset{N}{\underset{j=1\;}{\sum }}\overset{N-1}{\underset{i=1\&i\neq j}{\sum }}P\left(\;A_{i}\;\right)P\left(\;A_{j}\;\right)d_{ij}, \end{array}$$

where *P*(*A*_*i*_) and *P*(*A*_*j*_) stand for the probability of activities *A*_*i*_ and *A*_*j*_.

The bigger the feature's* AverageDivergence* is, the greater contribution to the separability of activities the feature has made. As* AverageDivergence* directly reflects one feature's distinguishing capability and has a linear relationship with classification accuracy, in this study, we take it as a standard for filtering features.

#### 3.3.2. Feature Selection

In this study, 50 features that are widely used in related articles [2–7, 13, 15] are chosen for candidate selection, like mean, variance, interquartile range, signal magnitude area (SMA), and so on. However, the number of features applied in one classifier is not the best. Feature selection can be realized from two aspects: (1) remove the useless features and (2) remove the related components. In order to better explain this problem, we propose a Divergence-based Feature Selection Algorithm (DFSA) on the basis of floating search method [18]. DFSA is detailed as follows.

Given a feature set that consists of *N* features (*N* = 50 in this paper), we aim to find a feature subset with the best *k*  (*k* = 1,2,…, *l* ≤ *N*) features resulting in the largest average divergence, namely, the best classification performance. Denote *X*_*k*_ = {**x**_1_, **x**_2_,…, **x**_*k*_} as the combination of the best *k* features and the rest of *N* − *k* features are denoted as *Y*_*N*−*k*_. We reserve all best subsets of low dimension *X*_2_, *X*_3_,…, *X*_*k*−1_, respectively, corresponding to 2,3,…, *k* − 1 features. The important functions *D*(•) are defined to present a feature's importance. For features in *X*_*k*_, *D*(•) is denoted as

(8) $$\begin{array}{c} D_{k-1}\left(\;x_{t}\;\right)=\bar{d}\left(\;X_{k}\;\right)-\bar{d}\left(\;X_{k}-x_{t}\;\right),\;if\;\;x_{t}\in X_{k}. \end{array}$$

For features not in *X*_*k*_, *D*(•) is denoted as

(9) $$\begin{array}{c} D_{k+1}\left(\;x_{t}\;\right)=\bar{d}\left(\;X_{k}+x_{t}\;\right)-\bar{d}\left(\;X_{k}\;\right),\;if\;\;x_{t}\notin X_{k}. \end{array}$$

In selected features set *X*_*k*_, the most important feature **x**_*t*_ is defined as the feature with the largest divergence contribution, subjecting to

(10) $$\begin{array}{c} D_{k-1}\left(\;x_{t}\;\right)=\underset{x_{t}\in X_{k}}{\max}\;D_{k-1}\left(\;x_{t}\;\right); \end{array}$$

the least importance feature **x**_*t*_ is defined as the feature with the smallest divergence contribution, subjecting to

(11) $$\begin{array}{c} D_{k-1}\left(\;x_{t}\;\right)=\underset{x_{t}\in X_{k}}{\min}\;D_{k-1}\left(\;x_{t}\;\right). \end{array}$$

Similarly, in candidate features set *Y* − *X*_*k*_, the most important feature **x**_*t*_ is defined as the feature with the largest divergence contribution, subjecting to

(12) $$\begin{array}{c} D_{k+1}\left(\;x_{t}\;\right)=\underset{x_{t}\in Y-X_{k}}{\max}D_{k+1}\left(\;x_{t}\;\right) \end{array}$$

and the least importance feature **x**_*t*_ is defined as the feature with the smallest divergence contribution, subjecting to

(13) $$\begin{array}{c} D_{k+1}\left(\;x_{t}\;\right)=\underset{x_{t}\in Y-X_{k}}{\min}D_{k+1}\left(\;x_{t}\;\right). \end{array}$$

The core of this algorithm is in the next step, by borrowing a feature from *Y*_*m*−*k*_ construct the (*k* + 1)th, key feature subset *X*_*k*+1_; then turn back to lower dimensional subsets to verify whether average divergence has been improved while new feature is added. If so, replace previously selected features with new one. To obtain the best feature subset to maximize the classification performance of each classifier, DFSA is described as shown in Algorithm 1.

## 4. Experiments and Analysis

This section describes detailed experimental setting and results that demonstrate the typical classification performance of RT-PKDT. Detailed comparison between RT-PKDT and several existing approaches (SVM, BP, and Bayesian Network) has been carried on to verify the applicability of RT-PKDT.

### 4.1. Experimental Setting

**Figure 7:**
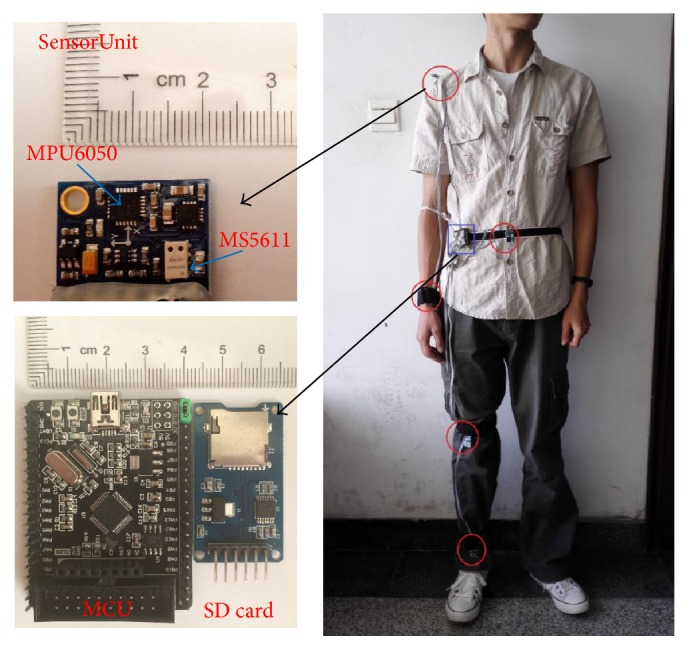
Experimental Platform Settings. Each sensor unit is mounted onto body locations tagged by red circles. MCU and storage unit is located in place marked with blue box.

Our activity recognition platform consists of five sensor units mounted to different parts of body listed in* Location* case set to collectively detect transitional movements listed in* activity* case set. Each sensor unit has a 6-axis sensor (MPU6050, which integrates a triaxial accelerometer and a triaxial gyroscope), and a barometer sensor (MS5611). The five sensor units are connected to a microcontroller (STM32F103) via cable wires for the sake of sampling efficiency in a rate of 10 Hz and data are recorded to SD card in real-time. The whole system architecture is demonstrated in Figure 7.

Experiments are conducted over the data set sampled by the above platform at 10 Hz. More than 30000 samples of each activity listed in* activity* set are taken and a 10-fold cross validation is applied to ensure that the sample set is large enough to guarantee the classification accuracy and generalization performance. We use the presented platform for data collection and perform all processing work offline in MATLAB with PC (Intel Core i5-3210M CPU, 8 G RAM). Our dataset is open sourced at https://github.com/Ethan–Xu/PKDT-dataset.

Furthermore, a publicly available dataset [9] is adopted for comparison to other approaches. In this dataset, a total of 16 people, 6 females and 10 males, aged between 23 and 50 years, of different height, weight, and constitution participated in the acquisition of the test data set. They were all asked to follow a schedule of which activities to perform and in which order, to allow us to cover all activities (containing all activities in* activity* case). Test candidates were asked to execute them in their personal style without a strict choreography. They even were encouraged to perform the same activities differently and to sometimes perform these activities in such way that a human observer could just about identify them accurately. Data were recorded in indoor and outdoor environment under seminaturalistic conditions. The sensor was placed on the belt of the test candidate either on the right or the left part of the body.

### 4.2. Results Analysis

To verify the validity of RT-PKDT on HAR problem, we take Support Vector Machine, BP neural work and Bayesian Network algorithms which are the most widely used algorithms in the study of HAR to make a brute-force comparison. We used the experimenter environment in the WEKA toolkit, with or without transition taken into consideration.

A radial basis kernel (RBF) based SVM is adopted using LibSVM [17] with automatic parameter selection through grid searching techniques. For the BP neural work, we take the standard approach of recursively evaluating values for the learning rate adopted in [12] and momentum using cross validation. Method described in [7] is applied as a typical Bayesian Network example. A 10-fold cross validation is applied to each classifier independently and the experiment results are shown in Table 2. From Table 2 we can see that the four algorithms show different classification accuracy on both data sets.

According to the performance, on collected data set, they can be sorted in the following order: RT-PKDT>BayesianNetwork>SVM>BP. Furthermore, RT-PKDT shows the highest global average classification accuracy reflecting a high stability during the classification. Similar performance are also presented on public data set [9]. In each independent activity, RT-PKDT also presents a better performance in classification accuracy and stability. SVM and Bayesian Network present similar effectiveness but they both show badly consistency on the recognition accuracy of different motions. For some specific human motion, the accuracy is rather low. They did not perform well as some of the testing activities may have similar feature distribution leading to fuzzy boundaries in the classification process. It may be because the training of multilayer perceptron is relatively complicated in this recognition problem and leads to overfitting. Besides, the long-time consumption in training phase of BP makes it unfit for real-time application.

For better comparison, Table 3 demonstrates the experiment results of several related works, using the methods of decision tree, *k*-NN, neural networks, and SVM. In contrast with self-designed algorithms used in Table 2, better results have been reached with improved ones in these related works, and particularly in [8] accuracy has been as high as 93.3%. However, our proposed RT-PKDT method still stands out with a highest accuracy 96.68%. Besides, RT-PKDT makes the advantage of motions' physical attributes which makes it more readable and easy to be understood and at the same time improves the classification performance with temporal information taken into consideration.

### 4.3. Comparison with Deep Learning Method

Apart from the methods mentioned above, deep learning is a hotspot of current research. Deep learning refers broadly to a branch of machine learning based on a set of algorithms that attempt to model high-level abstractions in data by using a deep graph with multiple processing layers, composed of multiple linear and nonlinear transformations. Deep learning techniques have outperformed many conventional methods in computer vision and audio classification. On human motion recognition issue, some related research has been done. For example, Ordóñez and Roggen [19] proposed a generic deep framework (DeepConvLSTM) for activity recognition based on convolutional and LSTM recurrent units. LSTM can also make use of temporal information which is stressed through this article. The DeepConvLSTM is evaluated on two public activity recognition datasets and the accuracy is around 90%.

However, problems exist that deep learning method has a strong dependency on data size. Human motion related activity recognition can seldom meet the needs of this large amount of data. Contrast experiment is conducted on the data collected in this paper by DeepConvLSTM method. An accuracy of only 22% is achieved, comparing with 96.68% of RT-PKDT. Results show that deep learning method is not that fit to human motion recognition problem due to its data size dependency.

## 5. Conclusion

The major contribution of this work is the proposal of a knowledge-driven method to recognize motion related human activities. In this study, we construct a conceptual model of motion related activities with exploring common domain knowledge with taken temporal information into consideration. RT-PKDT can be viewed as a recognition method with knowledge applied into the dealing of data which at the same time covers the advantages of data-driven methods. With a set of hierarchical rules successively applied to the recognition process, RT-PKDT shows a better recognition accuracy (96.68% on average). Compared with other algorithms, our proposed HPKDT method has the highest classification accuracy as well as a rather high efficiency. The efficiency of RT-PKDT is contributed by the following three factors. The first factor to promote classification accuracy is the deep analysis of different activities' attributes which concentrated features can far more embody the differences. The second factor to improve performance is making the most of temporal dependencies of human motions. Besides, a feedback method is adopted via fixing the estimated result at time *t* with result at time *t* + 1. The recurrent transition relationship among motions uses the temporal information to the max extent. RT-PKDT enhances classification performance with introducing knowledge into classifier and bringing in a set of hierarchical rules which are successively applied to the input data. All above reasons contribute to RT-PKDT's outstanding performance.

## Figures and Tables

**Algorithm 1:**
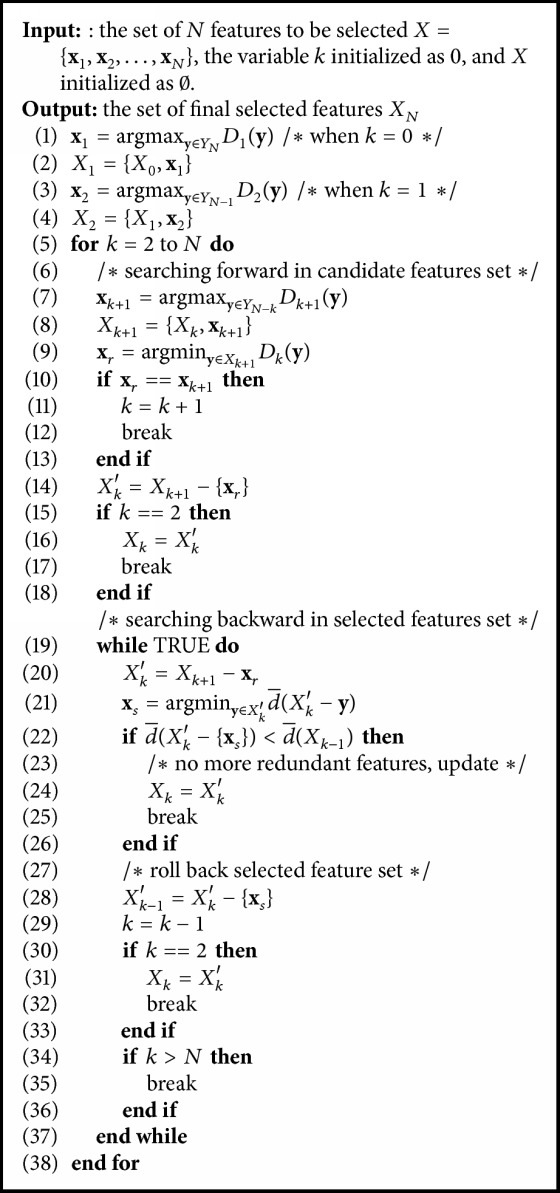
Divergence-based Feature Selection Algorithm.

**Table 1:** Possible Transitions between time *t* − 1 and time *t*. The first-order transition matrix is denoted as Trans1(*t* − 1, *t*).

Trans1(*t* − 1, *t*)	St[*t* − 1]	Ly[*t* − 1]	Eu[*t* − 1]	Ed[*t* − 1]	Up[*t* − 1]	Do[*t* − 1]	Wa[*t* − 1]	Ru[*t* − 1]	Tu[*t* − 1]
St[*t*]	1	0	1	1	0	0	1	0	1
Ly[*t*]	0	1	0	0	0	0	0	0	1
Eu[*t*]	1	0	1	0	0	0	1	0	0
Ed[*t*]	1	0	0	1	0	0	1	0	0
Up[*t*]	0	0	0	0	1	1	1	0	0
Do[*t*]	0	0	0	0	1	1	1	0	0
Wa[*t*]	1	0	1	1	1	1	1	1	0
Ru[*t*]	0	0	0	0	0	0	1	1	0
Tu[*t*]	1	1	0	0	0	0	0	0	1

**Table 2:** Classification accuracy (%).

	On collected data set				On public data set [9]			
	SVM	BP	BayesianNet	RT-PKDT	SVM	BP	BayesianNet	RT-PKDT
Standing	97.71	96.90	94.67	**99.39**	92.76	95.55	89.35	**97.22**
Lying	**100**	99.88	98.56	**100**	**99.85 **	99.88	98.55	99.63
ElevatorUp	92.37	99.15	**99.58**	94.49	90.33	93.55	**97.23**	96.21
ClevatorDown	88.44	83.56	**97.33**	93.78	90.98	91.12	92.88	**94.44**
Upstairs	94.12	18.82	81.18	**98.82**	93.32	89.56	84.88	**96.55**
Downstairs	83.1	69.01	83.10	**95.77**	89.55	78.43	88.21	**94.66**
Walking	**95.12**	90.14	96.14	91.87	**96.22**	92.98	91.33	93.22
Running	99.16	48.74	100	**100**	98.84	82.35	98.32	**99.35**
Turning-St-Ly	84.00	58.67	84.00	**96.00**	88.76	75.35	86.35	**90.05**

Average accuracy	92.67	73.87	92.73	**96.68**	93.40	88.75	91.90	**95.70**

**Table 3:** Comparisons with methods in other literatures.

Method	Candidate motions	Sensors type	Sensors location	Accuracy
Decision tree [8]	25 actions, Stand-Sit, Sit-Lie, etc.	Accelerometer, gyroscope	9, wrist, arm, ankle, etc.	93.3%
*K*-NN [10]	25 actions, Stand-Sit, Sit-Lie, etc.	Accelerometer, gyroscope	8, waist, left-forearm, etc.	92.2%
Neural Networks [11]	12 actions, Standing, Lying, etc.	Accelerometer	5, left forearm, trunk, etc.	89.2%
SVM [12]	8 actions, running, upstairs, etc.	Accelerometer, gyroscope, Magnetometer, barometer sensor	1, hand	88.6%
Bayesian Network [7]	7 actions, running, walking, etc.	Accelerometer, gyroscope, Magnetometer	1, belt	90%
proposed RT-PKDT	8 actions, listed in *Activity*	Accelerometer, gyroscope, barometer sensor	5 body-positions, listed in *Location*	**96.68**%

## References

[1] Mao X., Li M., Li W. (2017). Progress in EEG-based brain robot interaction systems. *Computational Intelligence and Neuroscience*.

[2] Scheurer S., Tedesco S., Brown K. N., O'Flynn B. Human activity recognition for emergency first responders via body-worn inertial sensors.

[3] Yassine A., Singh S., Alamri A. (2017). Mining human activity patterns from smart home big data for health care applications. *IEEE Access*.

[4] Xu C., He J., Zhang X., Yao C., Tseng P. (2018). Geometrical kinematic modeling on human motion using method of multi-sensor fusion. *Information Fusion*.

[5] Stiefmeier T. (2008). *Real-time spotting of human activities [Ph.D. thesis]*.

[6] Poggio T., Mhaskar H., Rosasco L., Miranda B., Liao Q. (2017). Why and when can deep-but not shallow-networks avoid the curse of dimensionality: a review. *International Journal of Automation and Computing*.

[7] Frank K., Diaz E. M., Robertson P., Sánchez F. J. F. Bayesian recognition of safety relevant motion activities with inertial sensors and barometer.

[8] Ghasemzadeh H., Jafari R. (2011). Physical movement monitoring using body sensor networks: A phonological approach to construct spatial decision trees. *IEEE Transactions on Industrial Informatics*.

[9] Swain P. H., King R. C. Two effective feature selection criteria for multispectral remote sensing.

[10] Ghasemzadeh H., Guenterberg E., Jafari R. (2009). Energy-efficient information-driven coverage for physical movement monitoring in body sensor networks. *IEEE Journal on Selected Areas in Communications*.

[11] Wang Z., Jiang M., Hu Y., Li H. (2012). An incremental learning method based on probabilistic neural networks and adjustable fuzzy clustering for human activity recognition by using wearable sensors. *IEEE Transactions on Information Technology in Biomedicine*.

[12] Zhang H., Yuan W., Shen Q., Li T., Chang H. (2015). A handheld inertial pedestrian navigation system with accurate step modes and device poses recognition. *IEEE Sensors Journal*.

[13] Aliakbarpour H., Khoshhal K., Quintas J. (2011). HMM-based abnormal behaviour detection using heterogeneous sensor network. *Technological Innovation for Sustainability*.

[14] Xu C., He J., Zhang X., Wang C., Duan S. (2017). Detection of freezing of gait using template-matching-based approaches. *Journal of Sensors*.

[15] Bousquet O., Boucheron S., Lugosi G. (2004). Introduction to statistical learning theory. *Advanced Lectures on Machine Learning*.

[16] Chen L., Hoey J., Nugent C. D., Cook D. J., Yu Z. (2012). Sensor-based activity recognition. *IEEE Transactions on Systems, Man, and Cybernetics, Part C: Applications and Reviews*.

[17] Chang C., Lin C. (2011). LIBSVM: a Library for support vector machines. *ACM Transactions on Intelligent Systems and Technology*.

[18] Pudil P., Kittler J. (1994). *Floating search methods in feature selection*.

[19] Ordóñez F. J., Roggen D. (2016). Deep convolutional and LSTM recurrent neural networks for multimodal wearable activity recognition. *Sensors*.

